# Driving the quality of care in publicly financed health insurance: a close look at the accreditation-based incentive policy of AB-PMJAY in India

**DOI:** 10.3389/fpubh.2026.1940730

**Published:** 2026-10-19

**Authors:** Arif Raza, Grace Achungura, Kheya Melo Furtado, Jaidev Singh Anand, Nafisa Vaz, Supriya Phadnis

**Affiliations:** 1 Goa Institute of Management, Sattari, Goa, India; 2 World Health Organization, Country Office for India, New Delhi, India

**Keywords:** accreditation, health system quality, healthcare quality, outcome based care, publicly financed health insurance, AB-PMJAY, India

## Abstract

The quality of healthcare delivery, in terms of clinical service standardization and patient satisfaction, remains a persistent challenge for low and middle-income countries (LMIC), including India. Since the launch of the *Ayushman Bharat Pradhan Mantri Jan Arogya Yojana* (AB PMJAY)—the world’s largest publicly financed health insurance scheme in terms of the numbers of beneficiaries eligible for coverage—the government has undertaken several initiatives to integrate quality-related interventions into the services delivered by empaneled healthcare providers. Among these was the introduction of financial incentives to empaneled healthcare providers linked to the uptake of quality certifications and accreditations in 2019. This policy and practice review examines the design assumptions of the accreditation-linked incentive policy, its early implementation experience and the uptake of quality certification among healthcare providers. It draws on the findings of a mixed-methods study commissioned by the National Health Authority of India, conducted in 2021 and published as a technical report, and extends them with certification uptake data to September 2026. Uptake of certification has been low and concentrated among hospitals that were previously accredited through independent accreditation bodies; providers’ responses to the incentive have been varied; and no clear relationship between certification tier and patient satisfaction has been demonstrated, although higher tiers corresponded to better-standardised structures and processes in the facilities observed. We examine the design assumptions that may explain this pattern and suggest the conditions under which the policy could achieve its intended effects. We submit that while the current design is largely an input-driven mechanism to motivate facilities to improve quality standards, it may serve only as a foundational step. For comprehensive quality improvement, we submit that the policy must evolve towards a suitable value-based incentive structure, aligning with international best practices, while providing a value proposition to empaneled hospitals vis-a-vis investments required for accreditation. We suggest actionable recommendations to strengthen the existing policy, spanning value-based incentive design, methods for improving provider value proposition and cross-scheme alignment.

## 1 Introduction

In alignment with the global agenda of Universal Health Coverage (UHC), India’s National Health Policy 2017 commits to publicly financed access to secondary and tertiary healthcare services, while delivery may occur through the mixed public-private health system (1). As a key step towards this goal, the *Ayushman Bharat Pradhan Mantri Jan Arogya Yojana* (AB PMJAY), initiated in 2018, was considered a central policy strategy (2, 3). Centrally administered by the National Health Authority (NHA), the program provides financial protection for hospitalisation services of up to INR 500,000 (approximately USD 5,600) per eligible family per year, covering nearly 600 million individuals—around 45% of the country’s population—thus representing one of the world’s largest publicly funded health insurance (PFHI) programmes in terms of eligible beneficiaries (4). Poor and vulnerable families, identified on the basis of the Socio-Economic Caste Census of 2011, are eligible under the Central AB PMJAY program guidelines, with a scope for states to include additional population groups at their own expense.

While the scale and scope of AB-PMJAY represents a major effort to advance UHC in India, expanding coverage alone does not guarantee good health outcomes—the quality of care delivered is equally important and critical to the agenda of UHC (5, 6). Global evidence shows that low quality healthcare can be as detrimental as the lack of access (7–9). Policy makers in India have experienced significant challenges in expanding PFHI with adequate quality safeguards, as evidenced during the implementation of the *Rashtriya Swasthya Bima Yojana (RSBY, 2008–2018),* a predecessor programme to AB PMJAY, and in other recent evaluations of PFHIs. India’s past experience with PFHIs has highlighted critical governance challenges, including insufficient frameworks for ensuring and regulating the quality of care (10–12). In the absence of a robust quality-linked purchasing mechanism, there is always a risk that an increased utilization may not translate into improved health outcomes. Notwithstanding these challenges, strategies to mainstream quality across empaneled public and private health facilities were included in the design of AB PMJAY since the early phases of the scheme. One of the earliest strategies was the introduction of a hospital accreditation-based financial incentive policy to incentivize the adoption of quality standards within empaneled healthcare facilities.

This paper analyses the design and implementation of the accreditation-based incentive policy under AB-PMJAY. It makes an analytical contribution based on the findings of a mixed-methods field study on mainstreaming quality of care in empaneled hospitals under AB PMJAY, conducted in 2021, commissioned by the National Health Authority (NHA) and carried out by the World Health Organization (WHO) in collaboration with the Goa Institute of Management (13). The study was conducted to serve the needs of AB-PMJAY policymakers, who sought early evidence on uptake and provider response to the accreditation-based incentive strategy within the scheme. In this paper we identify three assumptions embedded in the design of the incentive policy—that accreditation reflects clinical quality, that patients have higher rated experiences in accredited hospitals, and that the financial incentive outweighs the cost of accreditation. We examine each assumption against the findings of the previously conducted study and the wider literature, extend the certification uptake data to September 2026, and discuss the implications of the current strategy within India’s move towards value-based purchasing. By doing so, the paper contributes insights on the implications of quality-linked financial incentives within large publicly financed health insurance schemes in an LMIC context and provides recommendations for strengthening the design and uptake of such policies going forward, in India with relevance for other LMICs.

Sections 1.1, 1.2 and 1.3 provide, respectively, an overview of the quality of care across India’s healthcare provider landscape, the role of accreditation within this context, and a brief account of the measures AB-PMJAY has adopted to address the quality of care. Section 2 describes the quality certification system and the accreditation-linked incentive policy under AB-PMJAY that this study reviews, together with the methods and data sources. Section 3 presents the results and analysis, bringing together the implementation experience and an assessment of each assumption on which the policy design rests. Section 4 discusses the findings, offers actionable recommendations and notes the key limitations, and Section 5 concludes.

### 1.1 Quality in Indian healthcare settings

India’s healthcare system caters to a substantial and heterogeneous population through a blend of public and private healthcare facilities. The tax-funded public healthcare facilities though cost-effective and geographically widespread, often suffer from deficits in the quality of infrastructure, adherence to clinical standards and patient-facing factors such as cleanliness and waiting times. This has partly contributed to the rise of private healthcare providers, which account for about 60.3% of all hospitalized cases (14). However, the private healthcare landscape exhibits considerable heterogeneity in scale, service portfolio, quality benchmarks, and pricing, ranging from a limited number of advanced tertiary care institutions offering premium services to mostly low-cost establishments operating with constrained infrastructure and potentially suboptimal conditions (15). The dichotomy of existence of few acclaimed institutions, pioneering innovative, cost-effective care coexisting with majorly substandard healthcare facilities providing unsafe services that disproportionately affect rural and socioeconomically vulnerable populations, has been earlier reported (7). The recent Lancet India Commission described system-wide quality deficiencies that are now the leading barrier to the achievement of UHC in India (16).

### 1.2 Relevance of health facility accreditation to quality concerns in Indian healthcare

As in most LMICs, accreditation in India is increasingly viewed as a key mechanism for bringing a degree of uniformity in service availability and ensuring consistent service quality across healthcare organisations (17, 18). Over the last decade, accreditation has witnessed growing adoption among private healthcare providers seeking to project themselves as organizations committed to delivering high-quality care. Despite its policy prominence, empirical evidence linking accreditation status to patient-level outcomes in the Indian healthcare context remains limited and mixed, necessitating careful examination of accreditation-linked incentive strategies.

The National Accreditation Board for Hospitals and Healthcare Providers (NABH) under the purview of the Quality Council of India, remains the most widely adopted accreditation certification in India, and is largely adopted by the private sector. Other accreditation providers operate in the market—each with distinct standards and frameworks—including the National Quality Assurance Standards (NQAS) used primarily in the public sector, Joint Commission International (JCI), and the privately promoted Quality and Accreditation Institute (QAI).

Despite multiple accreditation programs, India has a low share of accredited facilities—less than 2.5% in the public sector and an estimated 15% in the private sector (19, 20). Table 1 presents a brief overview of the accreditation landscape at the national level. These national level accreditation services are further supplemented by sub-national accreditation and condition-focused accreditation programmes.

**Table 1:** Healthcare accreditation landscape in India.

Accreditor	Initiation	Ownership	Accredited facilities as per type
National accreditation board for hospitals and healthcare providers (NABH)	2006	Quasi-government	Hospitals—1,558 Small Healthcare Organisations—1189 Blood Banks—149 Medical Imaging Services—317 Dental Healthcare facilities—497 AYUSH Hospitals (Ayurveda, Homeopathy, Unani, Siddha, Yoga and Naturopathy)—371 Eye Care Hospitals—587 Entry-level certified (HOPE)—1338 All—6,006
National quality assurance standards (NQAS)	2013	Government	District Hospitals—135 Sub-divisional Hospitals—40 Community Health Centres—88 Primary Health Centres—619 Urban Primary Health Centres—82 All—964
Joint commission international standards (JCI)	2005 (India)	Private (international)	Hospitals—54 Ambulatory care organizations—6 Others programs—3 All—63
Quality and accreditation institute (QAI)	2016	Private	Eye care centres—23 Hospitals—6 Small hospitals—7 IVF centres—61 Dialysis centres—8 Others ~ 23 All—128

Source—Author’s compilation based on data from official websites of the respective accreditation agencies (accessed: July 10, 2025).

### 1.3 Policies for quality of care under AB PMJAY

AB PMJAY was designed with several built-in measures to improve quality and prevent fraudulent behaviour. These include: more stringent hospital empanelment criteria drawing on the national accreditation standards (21), a robust anti-fraud framework, utilizing data analytics and audits to detect and mitigate fraud, (22). Standard Treatment Guidelines (STGs) across clinical treatment modalities, aiming to standardize and elevate the care processes within the AB PMJAY and ***accreditation-based incentive payments*** for empaneled healthcare facilities.

From among the policies undertaken to address healthcare quality concerns, we assessed the accreditation-based incentive policy—its design, early implementation and the plausibility of the assumptions underlying it with respect to standardisation of clinical and non-clinical care services and patient satisfaction—within the broader context of health system quality improvement. We present our observations and arguments on the policy design primarily based on the findings reported in the underlying technical report on mainstreaming quality of care in empaneled hospitals under AB PMJAY, conducted in 2021 (13).

## 2 Accreditation-based incentive policy under AB-PMJAY and study methods

### 2.1 Design of the policy and underlying assumptions

The accreditation-based incentive policy was launched as a part of AB PMJAY’s quality improvement framework in 2019. It introduced a tiered reimbursement structure for network healthcare providers, linking payment rates to their quality certification level, which, in turn, was determined by their accreditation type. This stratified system delineated network facilities into four distinct levels of quality, based on their accreditation, assigning variable reimbursement premiums above the baseline rate to each level. Key elements of this policy design are encapsulated in Figure 1.

**Figure 1:**
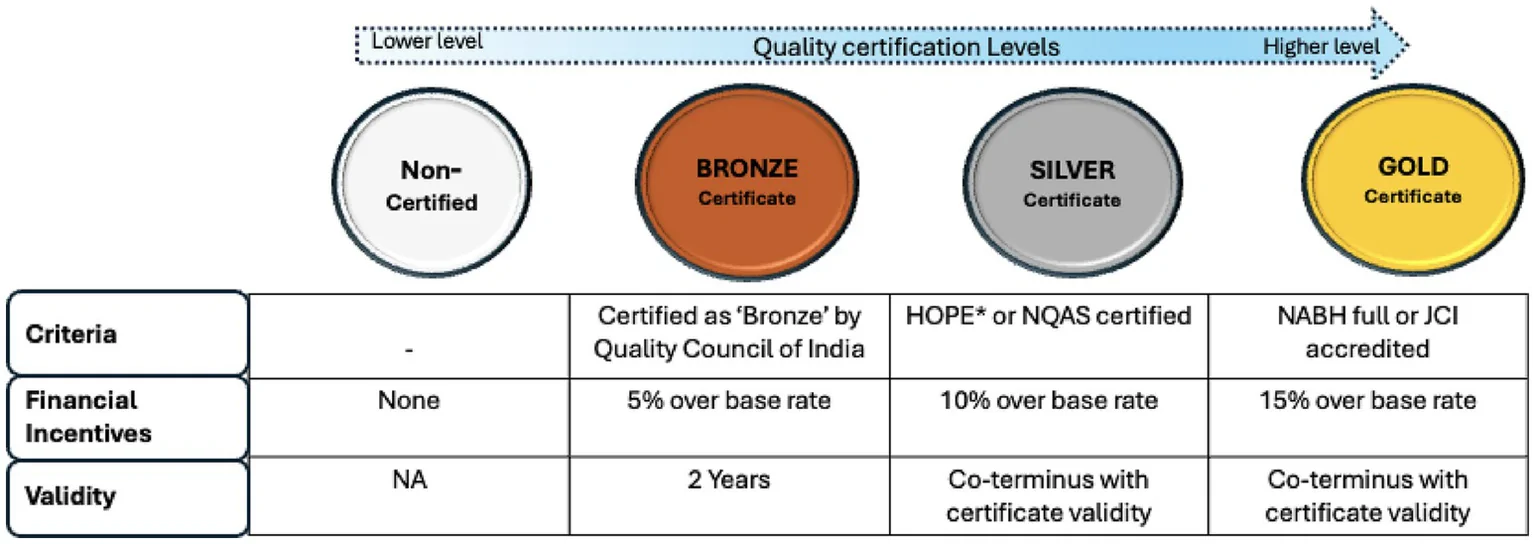
Accreditation-based incentive policy design. * Hospital organization platform for entry level certification—A temporary certification by NABH for healthcare facilities preparing for full accreditation. Source: author’s reconstruction based on a figure in the guidebook for AB PMJAY quality certification (23).

In this design, the gold and silver levels were aligned with existing accreditation programmes in the country, while the bronze was introduced as a new entry-level certification with relatively less stringent standards, intended to encourage non-accredited facilities to initiate their quality improvement journey and progressively advance along the accreditation continuum (23).

As per the policy document, accreditation-linked incentives aim to build a network of healthcare facilities delivering high quality, safe and patient-centred services, thereby enhancing patient satisfaction (23). For this, the policy design appears to be financially rewarding the healthcare facilities for enhancing their quality, albeit focused on improvement at input/structural level (accreditation status) rather than direct patient outcomes. The incentive structure is posited to attract accredited healthcare facilities to join AB PMJAY network and motivate existing network facilities to elevate their accreditation status as well as augment the treatment of AB PMJAY beneficiaries, in order to boost revenue. While administratively straightforward, this design equates accreditation status with quality, an assumption that requires closer scrutiny.

The design is underpinned by three implicit assumptions: *first*, it equates accreditation with improved clinical outcomes. *Second*, it further assumes that patients will have better experiences at accredited facilities, resulting in higher satisfaction. *Third*, a behavioural assumption is that healthcare facilities find the linked financial incentive attractive enough to improve their standards, and that the additional revenue from the incentives is sufficient to outweigh the costs of obtaining accreditation.

### 2.2 Methods and data sources

We examined the three assumptions underlying the design of AB-PMJAY’s accreditation-based incentive policy against the findings of a 2021 study and the wider literature, and extended the certification uptake data to September 2026 to assess how the strategy has progressed.

The above-referenced study was commissioned by the National Health Authority of India for the purpose of examining the early implementation experience of the quality certification system as described in section 2.1. This study was based on primary data collected through direct observation of 21 quality-certified private hospitals in Uttar Pradesh, Haryana and Gujarat (two districts each), sampled to represent certification tier (8 Bronze, 7 Silver, 6 Gold), bed size and rural/urban location and scored by a single trained team on a 62-item structure–process–outcome checklist, to evaluate the degree of service and infrastructure standardization. In addition, an analysis of 2,000 insurance claims was undertaken: a random sample of 1,000 claims each from 30 certified and 30 non-certified private hospitals across nine states, drawn from one month and sourced from the AB PMJAY claim management portal, compared on turnaround time, claim value and rejection using descriptive statistics and two-sample *t*-tests. In-depth interviews were conducted with the facility administrators of 29 of the 42 hospitals approached (all private; 8 non-certified, 8 Bronze, 7 Silver and 6 Gold, of which 8 were NABH fully accredited, 13 entry-level certified and 8 not accredited; 6 large, 16 medium and 7 small; 11 rural and 18 urban) and analysed thematically. Approximately 200 patients—beneficiaries discharged in the preceding 3 months from the 21 observed hospitals, of 300 sampled from lists supplied by the hospitals—were surveyed by telephone using the Patient Satisfaction Questionnaire Short Form (PSQ-18), with an added ten-point overall rating, enabling a structured, descriptive comparison between hospitals with NABH full accreditation, entry-level certification and no NABH accreditation (24). The certification uptake analysis drew on the complete NHA empanelment database (about 25,800 hospitals, of which about 12,300 private). Secondary data were compiled up to June 2021 and primary data were collected in July–August 2021. The samples for the observation, interview and survey components were purposive and small and their findings are treated throughout as early-implementation observations rather than as estimates of policy effect.

## 3 Results and analysis

We first describe the implementation experience of the quality certification system, including the system-level factors behind it, as evident from the underlying study, and then analyse the three implicit design assumptions in the light of that experience.

### 3.1 Implementation experience and uptake of accreditation among network hospitals

The following findings should be interpreted as early indicators of implementation dynamics rather than definitive measures of policy impact. In the initial years of the policy implementation, the uptake of the quality certification by network healthcare facilities, indicative of their interest in the accreditation-based incentive, has been limited and has not been sustained. As of September 2026, 380 healthcare facilities across India had obtained AB-PMJAY quality certifications (82 Bronze, 141 Silver and 157 Gold), constituting a small fraction—about 1.5%—of empaneled facilities (25).

At the time of the underlying study (June 2021), 508 facilities held a certificate—137 Gold, 206 Silver and 165 Bronze; (13) by September 2026 the number had fallen to 380, with Gold rising to 157 while Silver fell to 141 and Bronze to 82. Part of this pattern reflects the design of the tiers. Silver certification rests on NABH entry-level certification, which is valid for 2 years, during which the hospital is expected to prepare for and obtain full accreditation; a hospital that neither progresses nor renews loses its entry-level status and, with it, the Silver certificate. Bronze certification is likewise valid for 2 years and must be renewed to be retained. The decline in the Silver and Bronze tiers, set against a modest rise in Gold, is therefore indicative of limited interest among providers in acquiring, maintaining or upgrading their certification, and is consistent with the qualitative feedback from facilities on the certification system reported in Section 3.2.

The policy primarily leveraged existing accredited facilities, with most early adopters already pre-accredited. However, some large, fully accredited hospitals opted not to empanel under AB PMJAY, while smaller facilities showed early interest in Bronze and Silver certifications (13). Lack of awareness and the absence of a perceived need for it were the reasons given by hospitals that had not applied. Those that applied commonly did so because district or state officials asked them to. The Silver and Gold certificates confer no benefit beyond what NABH status already earns hospitals with other purchasers. Low uptake is therefore consistent with several mechanisms, and not only with the cost and revenue-share arguments set out in Sections 3.2 and 3.3.

### 3.2 System factors

The trend in quality certification—limited uptake and a net decline in the lower tiers—provides some insight into the interest of healthcare facilities, which the qualitative interviews help to explain. The qualitative in-depth interviews found that providers perceive significant systemic challenges within the broader AB PMJAY framework (13). Non-response was concentrated among non-certified hospitals, which is both a limitation and itself an indication of the level of interest in the policy.

A predominant concern relates to the inadequacy of current reimbursement rates under the programme, which reportedly constrains healthcare providers from delivering an optimal quality of care. These concerns have been reported repeatedly since the early days of the scheme and continue to persist, despite revisions to reimbursement rates over time (26). Delayed reimbursement was also reported as a concern, particularly by smaller facilities, thereby fostering scepticism regarding the reliability and timeliness of accreditation-related incentives. Delayed reimbursement under the programme has been reported by other studies on AB-PMJAY implementation (27). A few healthcare providers cited budgetary constraints as a limitation to pursuing higher levels of accreditation, which often involves a high cost. Most facilities with the higher accreditation levels indicated that their initial and ongoing accreditation efforts were driven predominantly by brand value considerations rather than the AB PMJAY financial incentives. Findings suggest a largely neutral stance among healthcare providers toward the incentive policy itself.

Overall, it is important to note that the purchasing power of the government remains limited through the implementation of AB-PMJAY and its associated state-specific schemes, which represent only about 3.4% of current health expenditure in 2022–23 (28). For a typical private hospital the scheme is therefore one revenue line among several, and a premium on that line is unlikely to outweigh accreditation costs unless AB-PMJAY volumes are high; the underlying study could not measure this exposure at facility level. Hence, such financial incentives are not likely to be sufficiently consequential to drive behaviour change among providers unless they extend beyond the scope of a single scheme.

### 3.3 Analysis of the implicit design assumptions

#### 3.3.1 Assumption 1: accreditation reflects clinical quality

While intuitively coherent, this assumption is scantily supported by empirical evidence, particularly within the Indian healthcare context. For instance, while some surveys among healthcare providers have shown that accreditation engenders a positive perception regarding patient safety and quality processes, these studies did not directly measure quality in terms of clinical outcomes (29, 30). While certain clinical procedures have shown improvement in outcomes post-accreditation (31), these findings may not be universally applicable to all procedures. The NABH accreditation system has faced criticism over its design limitations, with arguments that it reflects provider capability without guaranteeing the delivery of high-quality healthcare (32). The impact of JCI accreditation on healthcare quality, while documented as positive in other countries, (33) lacks data from the Indian healthcare context. Global literature also suggests that the relationship between accreditation and quality of care is not uniform across various healthcare settings, with effects fluctuating in magnitude and nature due to divergences in accreditation standards and the processes involved (34–38). In the underlying study, direct observation of 21 certified hospitals found that structure, process and outcome scores rose with NABH status: mean overall scores were 4.6 of 5 in fully accredited hospitals (*n* = 8), 3.5 in entry-level certified hospitals (*n* = 7) and 2.7 in hospitals without NABH accreditation (*n* = 6) (grouped by NABH status rather than by certification tier), with the least within-group variation among fully accredited hospitals; claims from accredited hospitals were also settled faster and carried higher values. Within the limits of a small purposive sample, the first assumption was therefore consistent with what was observed—accreditation tracked structural and process standardisation—although clinical outcomes as such were not measured.

#### 3.3.2 Assumption 2: patients experience accredited care as better

The assumption regarding patient experience presents similar uncertainties. A study in the state of Kerala indicated no significant difference in patient satisfaction between accredited and non-accredited hospitals (39). While accreditation standards, such as NABH’s requirements for monitoring patient rights and satisfaction metrics (e.g., waiting times, patient feedback), theoretically support enhanced patient experiences, satisfaction in healthcare remains inherently complex, influenced by human factors including empathy, responsiveness, and reliability (40). These intangible dimensions necessitate behavioural management strategies beyond mere structural compliance, which accreditation frameworks may not explicitly encompass. Additionally, for many private healthcare providers, AB PMJAY may represent a relatively less lucrative revenue stream compared to financially advantageous segments such as out-of-pocket payments (this forms the predominant source of revenue for private healthcare providers and contributes a large share of total health spending) and private health insurance, which may limit incentive to opt for the quality certification program. Consequently, providers may assign lower priority to AB PMJAY beneficiaries, which could adversely impact the satisfaction levels of these patients. In the beneficiary survey of the underlying study (*n* = 200; 74 patients treated in NABH fully accredited hospitals, 64 in entry-level certified hospitals and 62 in hospitals without NABH accreditation, all of which held an AB PMJAY quality certificate), mean overall hospital ratings on a ten-point scale were 7.9 (SD 2.8; 95% CI 7.3–8.5), 8.9 (SD 2.2; 95% CI 8.4–9.4) and 9.2 (SD 1.2; 95% CI 8.9–9.5) respectively. The same gradient appeared on all seven PSQ-18 sub-scales and in the proportion of respondents reporting a complaint (57%, 48 and 48%), even though the fully accredited hospitals had scored highest on the observed structure and process items. The underlying study reported these differences as not statistically significant. Several factors other than accreditation could account for the pattern: case mix and severity (fully accredited hospitals were larger and, in the claims analysis, submitted significantly higher-value claims); out-of-pocket payment, which was the most frequent complaint (47% of respondents) and more common among patients of fully accredited hospitals (54%); higher expectations of accredited hospitals; state effects; and the fact that respondent lists were supplied by the hospitals (13). The finding therefore does not show that accreditation lowers satisfaction, but it does indicate that the second design assumption cannot be taken for granted and warrants a dedicated, adequately powered study of patient experience across certification tiers.

#### 3.3.3 Assumption 3: the incentive outweighs the cost of accreditation

The third assumption—that the monetary incentive is large enough to outweigh the cost of accreditation— which was likely chosen based on what the programme could afford and what was deemed motivating, also needs examination. It is understandable that the level of influence of financial incentives on the strategic decisions of a healthcare facility will correlate to the proportion of revenue AB PMJAY contributes to their business. Additionally, accreditation entails significant costs—covering infrastructural upgrades, staff training, documentation, and accreditation fees; (41) potentially dissuading smaller facilities, unless AB PMJAY patient volumes justify such investments. The underlying study compared the direct cost of each tier with the premium it earns. Bronze, Silver and Gold certificates carry premiums of 5%, 10 and 15% over base package rates. No fee is charged by the NHA–QCI for the Silver and Gold certificates themselves, but they are available only to hospitals holding NABH entry-level certification or full accreditation, for which NABH charges a fee: INR 21,000 (small healthcare organisations) to INR 52,000 (hospitals) for entry-level certification, and, for full accreditation, an application fee of INR 25,000–1,50,000 plus an annual fee of INR 1,10,000–4,40,000 depending on hospital size, at 2021 rates. For hospitals that are already accredited, the marginal cost of certification is therefore close to nil; for others it is the full cost of accreditation, and the step from entry-level to full accreditation involves substantial infrastructure and staffing requirements (100 standards and 651 objective elements versus 45 and 167). Indirect preparation costs were not costed, and whether the premium covers them depends on each hospital’s AB PMJAY volume, which the study did not observe at facility level. The absence of explicit financial assistance for accreditation expenses within the policy further affects its operational viability. In the interviews, most facilities with higher accreditation levels attributed their accreditation to brand-value considerations rather than to the AB PMJAY incentive, and several had applied for the certificate because district or state officials asked them to. The evidence on the third assumption is thus indirect, but it points to a premium that is unlikely to be decisive for hospitals that are not already accredited.

Despite these concerns, the policy design exhibits notable operational strengths. Eligibility for each tier is a categorical, third-party-verified status—NABH accreditation level for Silver and Gold, and a QCI assessment for Bronze—that is periodically reassessed, which gives the scheme administrative simplicity and an objective criterion that is difficult to manipulate.

## 4 Discussion, actionable recommendations and limitations

### 4.1 Discussion

This analysis points to design limitations in, as well as the nuanced potential of, the accreditation-based incentive policy implemented under India’s AB PMJAY programme as a mechanism to drive healthcare quality. While the policy sensibly links financial incentives to accreditation status, which reflects structural quality, the assumptions in its design need careful examination. The modest interest and neutral attitude displayed by healthcare providers towards this policy, combined with their critical concerns with the broader AB PMJAY, are consistent with the design limitations identified in Section 3 and could undermine the objective of quality assurance and improvement.

In line with trends observed across several LMICs, India is progressively utilizing accreditation as a strategic mechanism to improve healthcare quality. A number of LMICs have linked accreditation to public purchasing in one form or another, and two cases illustrate a design contrast with AB-PMJAY (18). In the case of Indonesia, the implementation of the National Health Insurance scheme (Jaminan Kesehatan Nasional, JKN) significantly accelerated the adoption of accreditation, largely due to its embedded incentive structures, and the population-wide eligibility under JKN, requiring most hospitals to seek empanelment (42). In Ghana, accreditation by the National Health Insurance Authority is a legal requirement for any provider wishing to serve scheme members; of some 1,600 private providers assessed between 2009 and 2012, 78% were accredited, although most were graded in the lower categories and concerns were raised about post-accreditation monitoring (43).

In both schemes accreditation was a condition of participation in a purchaser covering most of the population, whereas AB-PMJAY made it a voluntary and marginal premium within a scheme that represents a small share of most private providers’ revenue. Moreover, such input-based incentive systems, including that of India, contrast with many globally endorsed quality improvement frameworks, which prioritize outcome-based measures of performance and patient care (44, 45).

A key criticism of being an input-focused strategy is that its impact on patient care outcomes could be limited and indirect. However, the accreditation-led model of India can be seen as a strategic foundational phase that prioritizes establishing essential structural standards first, creating a platform for future performance incentives. The strategy appears to be a pragmatic choice tailored to its context, designed to establish a baseline of quality as a crucial first step before advancing to more complex models.

Prospectively, India’s national health policy may need to transition towards outcome-centric frameworks to align with global standards. The draft policy on Value-Based Healthcare (VBHC) indicates this strategic pivot, proposing incentives linked to performance indicators like patient-reported outcomes and reduced re-admission rates (46).

This evolution is underpinned by the Ayushman Bharat Digital Mission (ABDM), which is architecting the integrated digital infrastructure necessary for a data-driven VBHC framework. The success of these policy measures and digital initiatives, however, hinges upon robust implementation and requires close assessment.

### 4.2 Actionable recommendations

Based on the above analysis and drawing from the literature, we suggest some actions to strengthen the adoption of quality certifications and accreditation within AB-PMJAY and comparable publicly financed health insurance schemes in LMICs. They are proposals grounded in the study findings and the wider literature rather than inferences from a representative sample.

The accreditation-linked incentive should be treated as a foundational phase and gradually evolve towards a more outcome-based framework to enhance quality assurance of healthcare under AB-PMJAY. Accreditation tiers can be retained as an eligibility floor, and the framework can be enhanced progressively. A robust outcome-based measurement framework should be established that identifies a list of outcome-based indicator name and ID; rationale/construct and dimension; standard formula for calculation; data source and collection method; risk adjustment method; reporting period; and target/benchmarks (47). A defined share of the incentive premium can then be shifted from accreditation tier to performance over the next few years, with risk adjustment by package and hospital size. This is a proposed direction of evolution, leveraging the robust information and communication technology systems for health financing developed under AB PM-JAY and the Ayushman Bharat Digital Mission (48).

The significant costs of accreditation can be offset through explicit financial or technical assistance, particularly for smaller facilities for which AB-PMJAY patient volumes alone may not justify the investment. The experience of Indonesia and Ghana suggests that uptake accelerates when accreditation is made a condition of participation and hospitals receive adequate financial support in the initial years; a phased move in this direction, with the entry-level Bronze certificate as the first step, could be considered as the purchasing share of AB-PMJAY grows.

The credibility of quality-linked incentives depends on the adequacy of reimbursement rates and the timeliness of payments. Continued revision of package rates and streamlining of claim settlement processes should therefore accompany the incentive policy.

Given that AB-PMJAY and its associated state-specific schemes represent only about 3.4% of current health expenditure in 2022–23, (28) quality-linked incentives should be aligned across public purchasing programmes to become sufficiently consequential to drive provider behaviour change. A practical first step is a common national quality-incentive schedule—a single, published mapping of accreditation tiers (and, in due course, performance indicators) to reimbursement premiums—adopted jointly by AB-PMJAY, CGHS, ESIC, the state employee schemes and other state-funded schemes, and offered to IRDAI-regulated insurers as a reference standard. The existing NHA–QCI certification registry could serve as the shared verification source, so that a hospital’s tier is assessed once and recognised by all public purchasers.

The uptake of quality certification and its relationship with clinical outcomes and patient satisfaction should be tracked over time, and a unit-level costing of accreditation relative to AB-PMJAY revenue should be undertaken, enabling iterative refinement of the policy design.

The digital infrastructure being built under ABDM also opens the way to analytical tools that could make outcome-based purchasing feasible at AB-PMJAY’s scale. The claims data analysed in the underlying study—turnaround times, claim values, rejections and package mix—are already generated for every admission; machine-learning models applied to the same data could flag anomalous billing and inappropriate utilisation, extending the scheme’s anti-fraud architecture, and could support risk adjustment by package and facility so that performance incentives do not penalise hospitals treating sicker patients. As clinical documentation moves onto ABDM-linked records, natural language processing could extract process and outcome indicators—adherence to standard treatment guidelines, complications, discharge status—without adding to the reporting burden on facilities, (49) and predictive models could identify patients at risk of readmission for targeted follow-up and more timely feedback to providers. Such tools should complement, not replace, clinical judgement, independent audit and patient-reported measures, and they carry well-documented risks: data quality and privacy, governance, interoperability, algorithmic bias, explainability and transparency, cybersecurity and legal accountability (50, 51). The uneven digital capacity between large private, small private and public hospitals—evident in the underlying study’s own observation and claims findings—means that an AI-enabled purchasing system introduced without technical and financial support would reward facilities for their data capability rather than for the care they provide. Clear data-governance standards, transparent validation, human oversight, continuous monitoring of algorithms and phased implementation are therefore preconditions if AI-enabled purchasing is to promote equitable quality improvement.

### 4.3 Limitations

The following methodological limitations should be considered while interpreting these results and recommendations. The observation, interview and survey components included in the underlying study were small and purposive samples, and hence the sub-group analyses were performed with very small denominators; survey respondents were drawn from lists supplied by the hospitals and could not be independently randomised; all primary data were collected in a single window in 2021, whereas the uptake figures have been updated to September 2026; clinical outcomes were not measured directly.

The uptake figures, which carry the central argument of the paper, come from the complete national empanelment database and the certification registry and are not subject to these sampling limitations, although the registry’s accuracy depends on the regularity with which it is updated by the National Health Authority.

## 5 Conclusion

Given the central role of AB PMJAY in India’s progress towards Universal Health Coverage (UHC), it is important to strengthen its quality component. The available evidence on the accreditation-based incentive policy supports three modest conclusions: uptake of quality certification among empaneled hospitals has been low and concentrated among hospitals that were already accredited; providers’ responses to the incentive are varied and, for most, shaped more by package rates, payment timeliness and brand considerations than by the premium itself; and no clear relationship between certification tier and either patient satisfaction or clinical outcomes has yet been demonstrated, although higher tiers did correspond to better-standardised structures and processes in the facilities observed. Existing measures—such as stringent empanelment criteria, anti-fraud mechanisms, standard treatment guidelines, and accreditation-linked incentives—primarily address the structural and process dimensions of care. While these interventions may positively influence health outcomes indirectly, a gradual shift toward an outcome-oriented framework is likely to be needed for more direct and sustained impact, with the accreditation-linked incentive treated as a foundation to be built upon rather than as a comprehensive instrument. In this regard, the draft Value-Based Healthcare (VBHC) policy constitutes a timely and necessary advancement. The recommendations in Section 4.2 are offered as initial proposals to strengthen the adoption of accreditation and quality certification among empaneled healthcare providers under AB PMJAY.

Future research should focus on the longitudinal assessment of this policy’s evolution including a unit-level costing of accreditation relative to AB PMJAY revenue, an adequately powered study of patient experience across certification tiers, and clinical outcomes for hospitals across certification tiers.
